# The impact of physical activity and exercise interventions on symptoms for women experiencing menopause: overview of reviews

**DOI:** 10.1186/s12905-024-03243-4

**Published:** 2024-07-13

**Authors:** Annemarie Money, Aylish MacKenzie, Gill Norman, Charlotte Eost-Telling, Danielle Harris, Jane McDermott, Chris Todd

**Affiliations:** 1 National Institute for Health and Care Research, Applied Research Collaboration Greater Manchester, School of Health Sciences, Faculty of Biology, Medicine and Health, The University of Manchester, Manchester, M13 9PL UK; 2 School of Health Sciences, Faculty of Biology, Medicine and Health, The University of Manchester, Manchester, M13 9PL UK; 3 Manchester University NHS Foundation Trust, Manchester, M13 9WL UK; 4 National Institute for Health and Care Research, Innovation Observatory, Newcastle University, Newcastle, NE4 5TG UK

**Keywords:** Menopause, Symptom management, Physical activity/Exercise, Quality of life, Overview of reviews

## Abstract

**Background:**

Women experiencing problematic menopausal symptoms report lower health-related quality of life and greater healthcare use than women without symptoms. Not all women want to or are able to take hormone replacement therapy. Strengthening the evidence for menopause symptom-management options, including physical activity, improves agency for women.

**Aim:**

This overview assesses effectiveness of physical activity and exercise interventions targeting women experiencing menopause symptoms.

**Methods:**

Medline, Embase, CINAHL, Scopus, The Cochrane Database of Systematic Reviews and Social Science Citation Index were searched (June 2023) for systematic reviews of physical activity and exercise interventions targeting women experiencing menopause. Reviews were assessed using AMSTAR-2 and a best-evidence approach to synthesis without meta-analysis (SWIM) was adopted. The protocol was registered on PROSPERO (CRD42022298908).

**Results:**

Seventeen reviews included 80 unique relevant primary studies with 8983 participants. There is evidence showing improvement of physical, urogenital, and total symptoms following yoga interventions. Evidence for vasomotor and psychological symptoms was inconclusive. Findings for aerobic exercise were inconclusive although there were some examples of beneficial effects on total and vasomotor symptoms. Evidence was very limited for other types of physical activity and impact on physical, sexual and urogenital symptoms.

**Conclusion:**

There is some evidence that yoga, and to lesser extent, aerobic exercise may be beneficial for some menopause symptoms, but there is insufficient evidence to recommend a particular form of exercise. Current reviews categorise women on menopause status; broadening this to include ethnicity, income status, employment and other factors will allow better understanding of context for successful interventions.

**Supplementary Information:**

The online version contains supplementary material available at 10.1186/s12905-024-03243-4.

## Introduction

The menopause - defined as cessation of menstrual periods - is a gradual, naturally occurring process for women that typically occurs between 42 and 55 years of age [1]. Problems ‘below the waist’ are often unattractive topics for public or political discourse; the UK Government’s recent publication of the Women’s Health Strategy [2] and establishment of the Menopause Task Force [3] have increased attention in the UK.

Many women (around 80%) experience sometimes debilitating symptoms associated with oestrogen and testosterone depletion for an average of four (but up to 12) years. These include hot flushes, night sweats, sleep disruption, fatigue, difficulty concentrating, depression and anxiety, mood swings, irritability, and loss of confidence [1]. Women experiencing problematic menopausal symptoms report lower levels of health-related quality of life and greater use of healthcare than women without symptoms. Prolonged lack of oestrogen impacts the cardiovascular system and can increase risk of long-term conditions including osteoporosis [4]. Although many women experience problematic menopause symptoms, many fewer use Hormone Replacement Treatment (HRT) [5].

Emerging evidence suggests physical activity may be beneficial [6] for alleviating some menopausal symptoms and helping prevent diseases with increased risk during menopause. Physical activity can be categorised into occupational, sports, conditioning, household, or other activities. Exercise is physical activity that is planned, structured, and repetitive and has improvement or maintenance of physical fitness as an objective [7]. There is strong evidence that, throughout life, physical activity is protective against chronic conditions including coronary heart disease, obesity, type 2 diabetes, and mental health problems [8]. UK recommendations are clear about the benefits of physical activity, importance of strength training, and amount of recommended weekly activity [9].

However, women may reduce physical activity during menopause transition [10]. In England 38% of women aged 45–54 years do less than the weekly recommended 150 min moderate activity, and 60% of adult women do less than twice weekly strength training [11]. The ‘Women in Sport’ report [10] found that, although physical activity levels were low, menopausal women’s desire to be more active was high, and increased when recommended by a health professional. Many women with menopause symptoms may seek medical advice from their GP. However, not all women want to, or are able to take, or access, HRT. Strengthening the evidence-base for all intervention options may improve agency over menopause symptom management for women [5].

## Aim

This overview aims to assess the effectiveness of physical activity and exercise interventions targeting women experiencing menopause. There are multiple recent and ongoing systematic reviews in this area, but no existing overview [12–14].

## Methods

The overview protocol was registered on PROSPERO (CRD42022298908) [15] which is an international prospective register of systematic reviews. This platform allows authors to view systematic reviews in progress and also improves accountability for authors to justify any decisions made to change their original published protocol. It was conducted using guidance from the Cochrane Handbook for Systematic Reviews of Interventions [16] and reported following the Preferred Reporting Items for Overviews of Reviews (PRIOR) [17].

### Inclusion criteria

The PICO (population, intervention, comparator, outcomes) inclusion criteria are provided in Box 1.

**Box 1:** Inclusion criteria for systematic reviews

**Population**	We included women with spontaneous or surgical menopause, in the peri-menopausal or postmenopausal period. Perimenopause is the interval in which a woman has irregular cycles of ovulation and menstruation before the menopause. Postmenopausal women are defined as those with surgical or spontaneous menopause and amenorrhoea for longer than 12 months.
**Interventions**	We included systematic reviews that evaluated physical activity or exercise interventions undertaken to reduce menopausal symptoms in women. Physical activity or exercise is defined as any bodily movement produced by skeletal muscles that results in energy expenditure. We placed no limits on how the activity is delivered and included both instructor-led or self-led programmes, accepting review authors’ definitions of activity. The types of interventions included, but were not limited to: Yoga; Resistance or strength training; Aerobic exercise; Walking; Flexibility or stretching training (including Pilates, Tai Chi, Qi Gong).
**Comparator**	We included systematic reviews that compared any type of exercise or physical activity with no active treatment, another type of exercise or physical activity, or with other treatments such as HRT as controls.
**Outcomes**	We included both menopause symptoms as a class, and specific types of symptoms. Types of menopause symptoms were broadly categorised as follows: vasomotor (hot flushes and night sweats), physical (musculoskeletal pain, headaches, palpitations, sleep problems, reduced muscle mass), urogenital symptoms (vaginal dryness), sexual difficulties (low sexual desire), psychological (low mood / anxiety, concentration problems) [1]. We also included a category for ‘total’ symptoms (i.e. vasomotor plus physical plus urogenital plus sexual plus psychological) which is the same term used in the MENQOL instrument [18, 19] and includes quality of life. We considered all symptom outcomes to be equally important. Improvement in menopause symptoms had to be assessed by one or more of a range of validated tools for both the intervention and the control groups. These could be generic or menopause specific instruments and included the following: Global QoL [20]; 36-item Short Form Health Survey (SF-36) [21]; World Health Organization QoL [22]; Utian QoL [23], Menopause-specific QoL [18, 19]; International Incontinence questionnaire [24]; Sexual Activity questionnaire [25]; Greene Climacteric scale [26]; Women’s health questionnaire [27]; Hot Flash Related Daily Interference Scale [28].

#### Types of studies

We included systematic reviews (with or without meta-analysis). Systematic reviews were defined using criteria from the Database of Abstracts of Reviews of Effects [29]. Reviews had to have: clear inclusion/exclusion criteria; adequate search strategy; synthesis of studies; and quality assessment of studies. Reviews reported only as conference abstracts, or not published in English, and review protocols were excluded.

#### Search strategy

In June 2023 we searched Medline, Embase (both OvidSP), CINAHL (EBSCO), Scopus (Elsevier), The Cochrane Database of Systematic Reviews and Social Science Citation Index (Clarivate); reference lists were also searched. These databases were chosen based on previous reviews on a similar topic. Date limitation (2000 onwards) was used because a Medline search produced no prior results and because validated tools and instruments were not previously routinely used for menopause symptoms [30]. The strategy combined keywords relating to physical activity, exercise, menopausal symptoms and study design (see Supplementary Table 3 for example search strategy). Only reviews written in the English language were included.

#### Selection criteria

Two independent reviewers (AM and DH/AMK) used the systematic review organisational web tool, Rayyan [31] to review both abstracts and full text articles of potentially relevant studies. This ensures robust screening of papers and reduces the risk of bias as papers are blinded until screening is complete. At each stage disagreements were resolved through discussion with a third reviewer (CET).

#### Data extraction and management

A data extraction table was created based on Cochrane guidance for overview of reviews [16]. Two reviewers (AM and AMK/DH) extracted data including: review methods and characteristics; participants and interventions; outcome data; and demographic characteristics in line with PROGRESSplus which is a validated list of characteristics known to affect health outcomes and equity [32]. PROGRESS refers to Place of residence; Race/ethnicity/culture/language; Occupation; Gender/sex; Religion; Education; Socioeconomic status; Social capital and PLUS refers to: (1) personal characteristics associated with discrimination (e.g. age, disability) (2) features of relationships (e.g. smoking parents, excluded from school 3) time-dependent relationships (e.g. leaving the hospital, respite care, other instances where a person may be temporarily at a disadvantage).

#### Methodological quality

Two reviewers (AM and AMK) independently assessed reviews using the AMSTAR-2 [33] (Assessing the Methodological Quality of Systematic Reviews) tool to generate overall ratings of ‘high’, ‘moderate’, ‘low’ or ‘critically low’ based on 16 questions/items, of which seven are deemed critical. The review team discussed critical domain weaknesses to produce consensus judgements. This tool was chosen as it is appropriate for systematic reviews that include both randomised and non-randomised trials.

#### Overlap

To assess overlap in included primary studies between reviews, the corrected covered area (CCA) [34] was calculated using GROOVE (Graphical Representation of Overlap for Overviews). This software calculates the percentage of studies that appear in two reviews. This was to ensure primary studies were not counted more than once which would potentially give false weighting to a result. A CCA score or > 15% indicates very high overlap.

#### Synthesis

We used an approach guided by the SWiM [35] (synthesis without meta-analysis) methodology, and key aspects of this approach are as follows. We considered two main types of comparison: physical activity versus no intervention and comparisons between two different types of activity. The synthesis was further grouped by activity type and then by symptoms. We used a best-evidence approach to focus on reviews with high-quality AMSTAR-2 ratings published within the past 10 years. Guided by GRADE domains, we also considered risk of bias, imprecision and inconsistency of evidence within each review and overall [36].

## Results

**Fig. 1:**
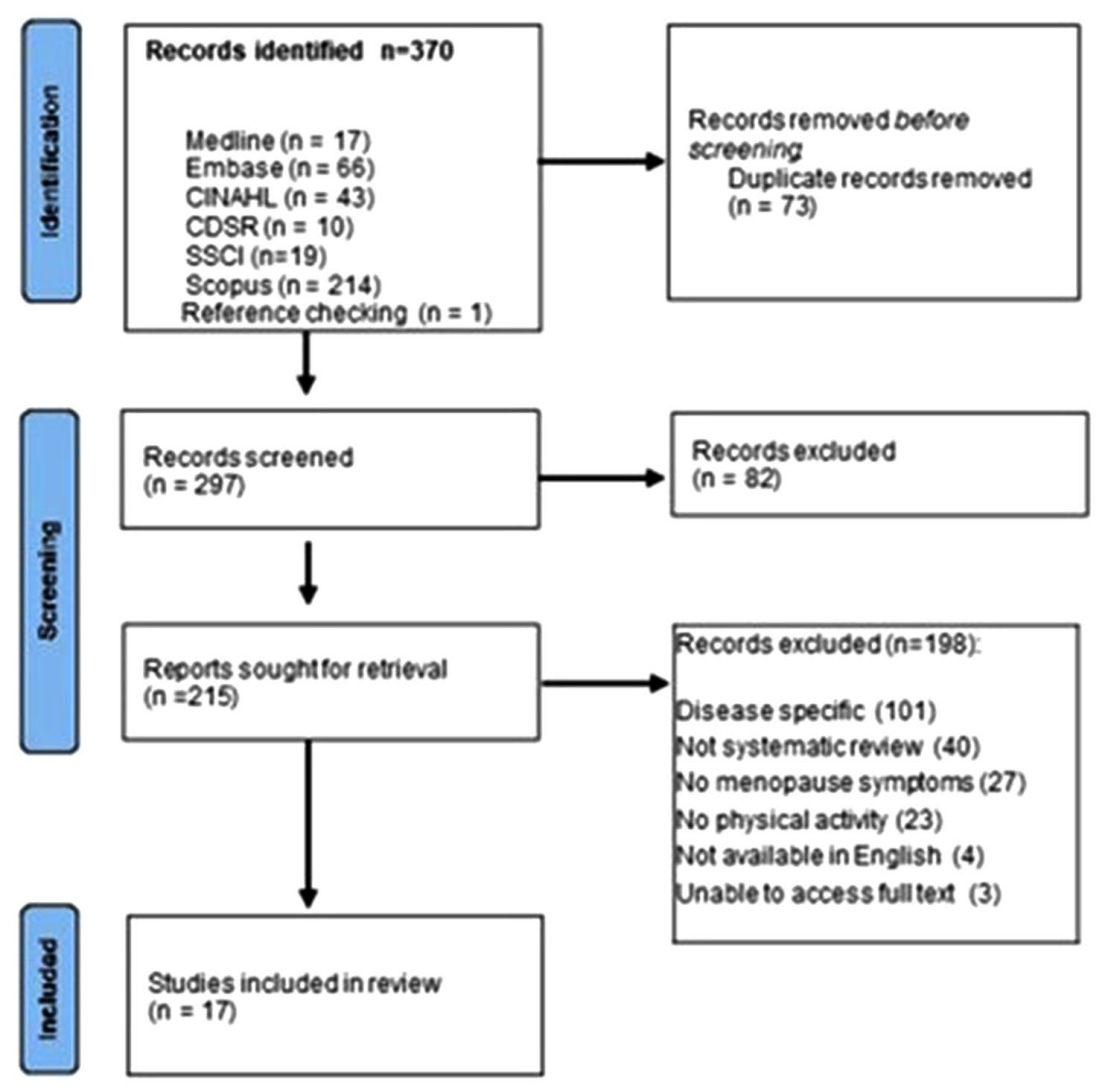
PRISMA flowchart of data selection

We identified 370 records (Fig. 1). Following deduplication, we assessed 297 abstracts and 215 full texts, and included seventeen systematic reviews [6, 14, 37–51].

### Description of included reviews

**Fig. 2:**
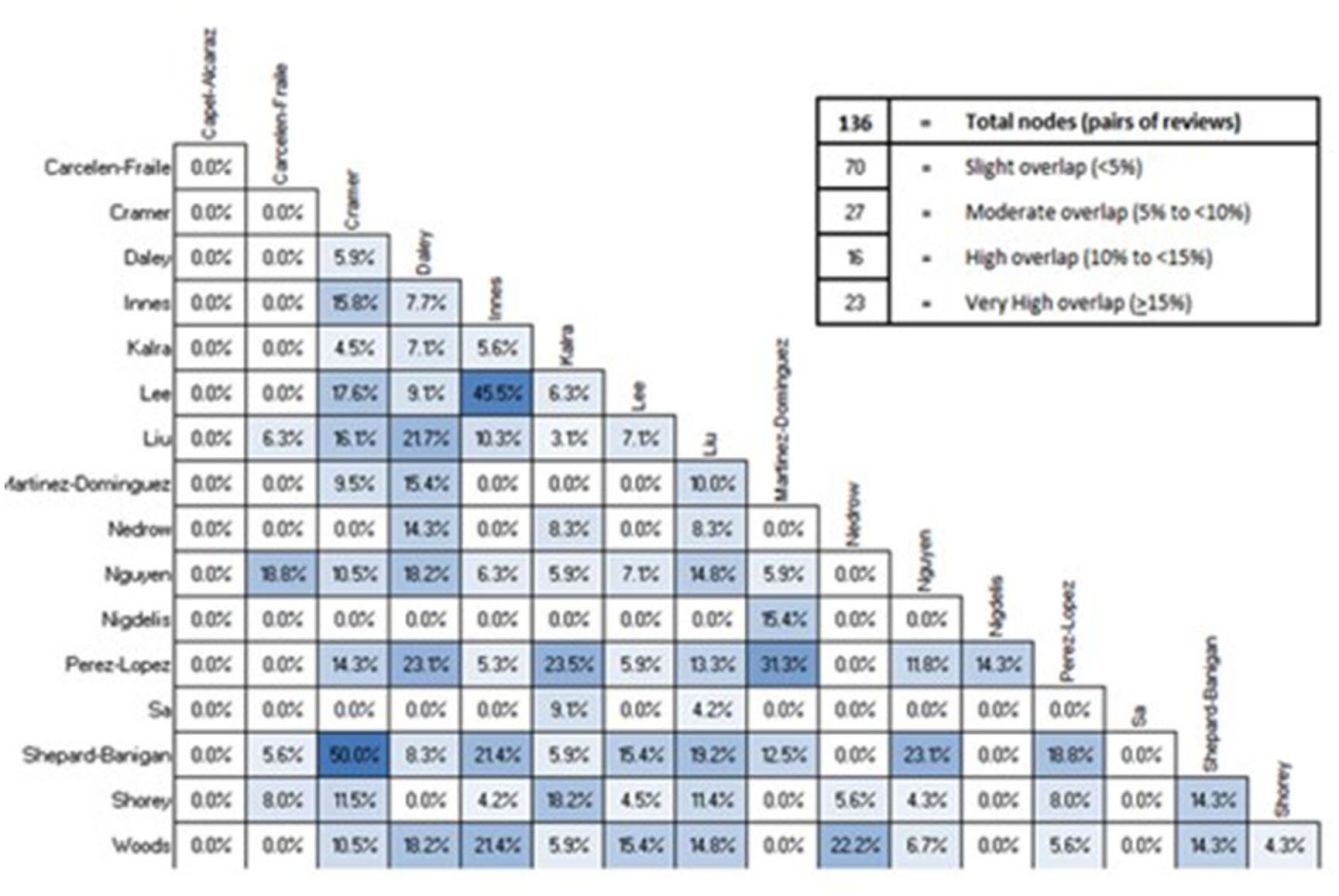
Corrected Coverage Area (CCA) for included reviews

Main review characteristics are presented in Table 1. From these reviews we extracted 80 unique studies (71 RCTs) and 8983 participants once overlap has been accounted for; study overlap is shown in Fig. 2. Reporting on demographic characteristics in line with PROGRESSPlus [32] guidance was limited.

**Table 1:** Summary of included systematic reviews

Author/Year	No. of studies included in overview* (Population)	Intervention type	Duration / Frequency / Session Length	Symptom category	AMSTAR2
Carcelen-Fraile et al. (2020) [37]	11 studies: 8 RCTs, 1 feasibility, 1 prospective, 1 pre-post study (1548)	Pelvic floor muscle training (PFM) Aerobic exercise Yoga Mind-body therapy.	Duration 12 weeks (range = 8 weeks - 24 months) Frequency: 1-5 times per week Length: 30–120 min	Total Vasomotor Physical Sexual	Low
Capel-Alcaraz et al. (2023) [49]	4 RCTs (274)	Resistance training	Duration: 12 – 52 weeks Frequency: 1-3 times per week Length: 55–60 min	Vasomotor Total	Low
Cramer et al. (2018) [38]	13 RCTs (1306)	Yoga	Duration: 4–16 (median 12) weeks, Frequency: 1–14 (median 2) times per week, Length: 20–120 (median 60) minutes per session	Total Vasomotor Psychological Physical Urogenital	High
Daley et al. (2014) [6]	5 RCTs (733)	Aerobic exercise	Duration: 3-6 months Frequency: 3-7 times per week Length: 40–60 min	Vasomotor	High
Innes et al. (2010) [39]	9 studies: 3 RCTs, 5 uncontrolled clinical trials, 1 nonrandomised controlled trial (484)	Mind-body therapy Yoga	Duration: 16-week programme (Tai Chi) Frequency: 1/2 - 5 sessions/week Length: 60–150 min	Total Vasomotor Psychological Physical	Moderate
Kalra et al. (2022) [40]	10 RCTs (833)	Aerobic exercise Yoga Resistance training Mind-body therapy	Duration: 6-52 weeks Frequency: 2-3 per week Length: 40–60 min	Total Psychological Physical	Critically low
Lee et al. (2009) [41]	7 studies, 3 RCTs, 1 controlled clinical trial, 3 uncontrolled clinical trial (470)	Yoga	Duration: 8-12 weeks Frequency: 2-3 times per week Length: 60–90 min	Total Vasomotor Psychological Physical	Low
Liu et al. (2022) [51]	21 RCTs (2884)	Aerobic exercise Yoga Resistance training Mind-body therapy	Duration: 3-48 weeks Frequency: 1-7 sessions a week Length: 30–120 min	Vasomotor	High
Martinez-dominguez et al. (2018) [48]	10 RCTs (1463)	Yoga Aerobic exercise Resistance training Moderate-high intensity Low intensity Medium-term Long-term	Duration ranged from 12 weeks to 14 months. ‘Mid-term exercise intervention’ (MTEI) = 12 weeks - 4 months ‘Long-term exercise intervention’ (LTEI) = 6-14 months	Psychological	High
Nedrow et al. (2006) [42]	3 RCTs (284)	Aerobic exercise	Duration: 6-52 weeks Frequency: 3 per week or 225 min per week. Length: Not reported	Total Vasomotor Psychological Physical	Moderate
Nguyen et al. (2020) [14]	9 RCTs (882)	Pelvic floor muscle training (PFM) Yoga Aerobic exercise	Duration: 4 weeks – 6 months, Frequency: 1-7 times per week Length: 20–90 min	Total Vasomotor Psychological Physical Sexual Urogenital	High
Nigdelis et al. (2018) [43]	5 RCTs (2112)	Aerobic exercise Resistance training Mind-body therapy Medium-term exercise Long-term exercise Moderate-high intensity Low intensity	Duration: 6 - 24 months Frequency: 3-5 per week Length: 30–60 min	Vasomotor Psychological Physical	Critically low
Perez-Lopez et al. (2017) [44]	11 RCTs (1943)	Medium-term exercise Long-term exercise	Duration: 6 weeks - 12 months (MTEI = 12 weeks - 4 months & LTEI = 6-12mths) Frequency: 2-5 per week Length: 40–90 min	Psychological	Low
Sa et al. (2022) [50]	2 RCTs (94)	Resistance training Aerobic exercise	Duration: 8 weeks & 15 weeks Frequency: 3 per week Length: Not reported	Vasomotor	High
Shepherd-Banigan et al. (2017) [45]	8 RCTs (927)	Yoga Mind-body therapy	Duration: 10-13 weeks Frequency: 1-7 per week Length: 15–90 min	Vasomotor Psychological	High
Shorey et al. (2020) [46]	15 RCTs (1337)	Mind-body therapy Aerobic exercise Resistance training Yoga	Duration: 1-24 weeks Frequency: Not reported Length: Not reported	Total Vasomotor	High
Woods et al. (2014) [47]	7 RCTs (1056)	Yoga Mind-body therapy Aerobic exercise	Duration: 3 weeks - 12 months Frequency: 1-5 weeks Length: 20–60 min	Vasomotor Psychological Physical	Critically low

*Prior to overlap correction (CCA)

Interventions included: aerobic exercise (11 reviews); yoga (11 reviews); other forms of mind-body therapy, such as stretching and relaxation, Tai Chi and Qi Gong (eight reviews); resistance/strength exercises (seven reviews); and pelvic floor muscle training (two reviews). Yoga is categorised separately to mind-body therapy due to the large number of reviews focusing solely on yoga. Intervention durations varied from one week to 24 months. Session length ranged from 20 to 120 min and frequency from 0.5 to 7 times per week. Comparators included: no active treatment, wait-list controls, health education or alternative exercise. Two RCTs in the reviews used HRT as a comparator but this was not reported at review level. Table 2 shows assessed comparisons and symptoms reported.

Eight reviews were rated high quality [6, 14, 38, 45, 46, 48, 50, 51] via AMSTAR-2, the remainder were moderate [39, 42], low [37, 41, 44, 49] or critically low quality [40, 43, 47] (Supplementary Table 2). The most common issues were lack of protocol before conducting the search, not justifying exclusion decisions, and not considering bias assessment in discussion of individual results. We summarise only the high-quality effectiveness evidence here; detailed results including effect estimates are in Table 3. Detailed results of moderate, low, and critically low-quality evidence are summarised in Supplementary Table 1.

**Table 2:**
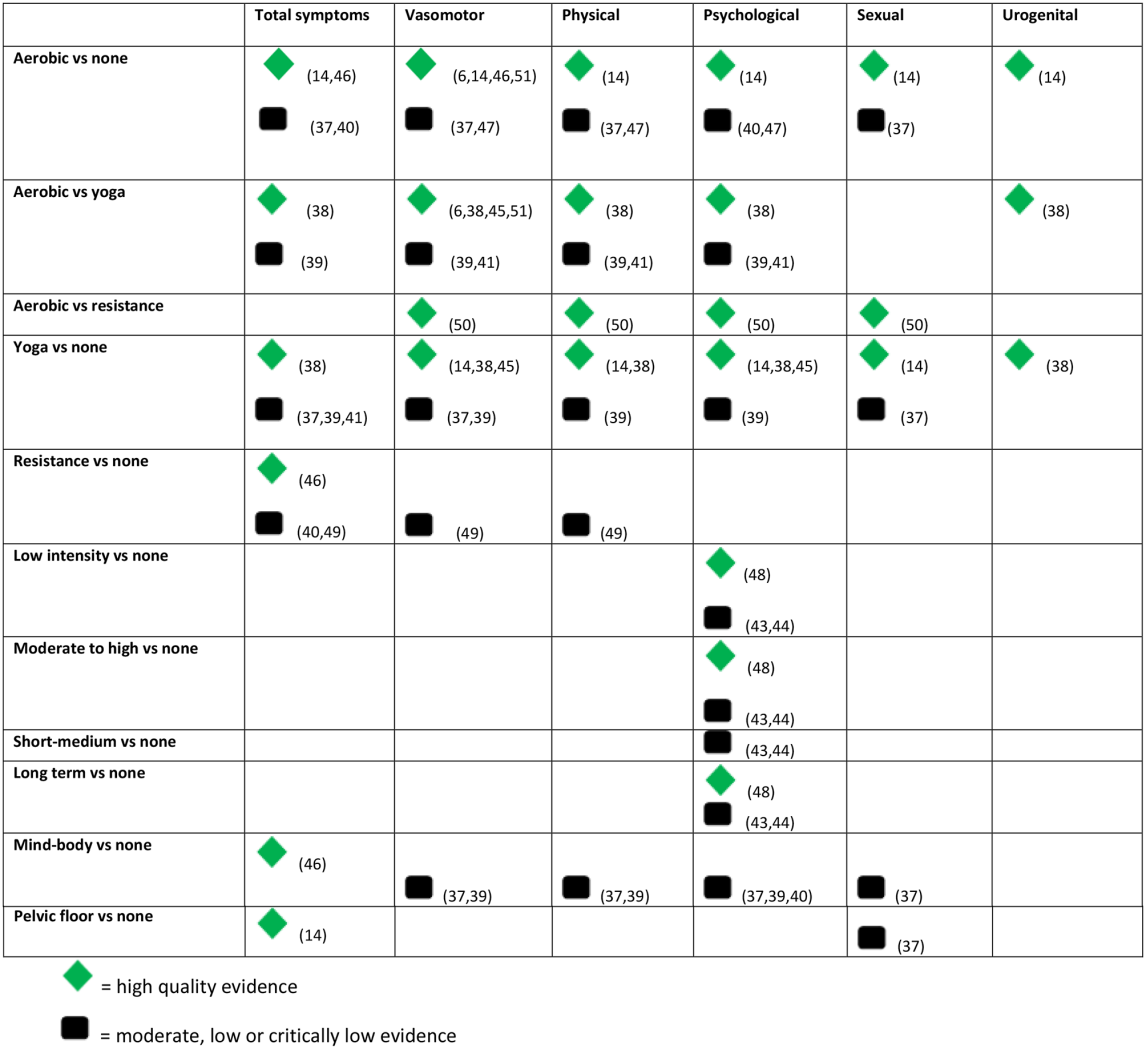
Evidence map by symptom category and comparison

**Table 3:** Summary of findings (high quality reviews only)

Outcome	Review	SMD	95%CI	I²	RCTs	Participants	ROB			
							High	Low		Unclear
**Aerobic exercise vs. no intervention**										
Total	Nguyen	0.23	-0.1 to 0.56	59%	5	441	1	X		4
	Shorey	-0.69	-1.09 to –0.28	0%	2	101	X	1		1
Vasomotor	Daley	-0.10	-0.33 to 0.13	30%	3	454	1	X		2
	Nguyen	-0.14	-0.42 to 0.15	63%	5	602	1	X		4
	Shorey	-0.26	-0.70 to 0.17	76%	4	358	X	2		2
	Liu (Frequency)	0.14	-0.03 to 0.31	35%	7	940	4	2		10
	Liu (Severity)	0.25	0.04 to 0.47	72%	11	1425	4	2		10
	Liu (Severity index)	0.67	0.17 to 1.17	46%	3	127	4	2		10
Psychological	Nguyen	0.56	-0.04 to 1.15	93%	6	812	1	X		5
Physical	Nguyen	0.89	-0.11 to 1.89	97%	5	660	1	X		4
Urogenital	Nguyen	-0.79	-1.92 to 0.34	85%	2	204	X	X		2
Sexual	Nguyen	-0.19	-0.43 to 0.04	36%	4	494	1	X		3
**Low intensity exercise vs. no intervention**										
Psychological	Martinez-Dominguez	-0.58	-1.39 to 0.24	93%	4	460	X	2		2
**Moderate to high intensity exercise vs. no intervention**										
Psychological	Martinez-Dominguez	-0.06	-0.22 to 0.09	41%	7	1250	X	X		7
**Short to medium term exercise vs. no intervention**										
Total	Shorey	-0.70	-1.22 to -0.18	76%	5	282	X	2		3
Psychological	Martinez-Dominguez	-0.42	-0.81 to -0.02	87%	7	939	X	2		5
**Long term exercise vs. no intervention**										
Psychological	Martinez-Dominguez	-0.03	-0.18 to 0.13	29%	5	1013	X	X		5
**Yoga vs. no intervention**										
Total	Cramer	-1.05	-1.57 to -0.53	88%	8	671	Bias not reported at primary study level.			
Vasomotor	Cramer	-0.76	-1.27 to -0.25	85%	8	548	Bias not reported at primary study level.			
	Nguyen	-0.37	-1.15 to 0.4	82%	3	179	Bias not reported at primary study level.			
	Shepherd-Banigan	-0.34	-0.92 to 0.25	0%	3	204	Bias not reported at primary study level.			
Psychological	Cramer	-0.75	-1.17 to -0.34	84%	10	756	Bias not reported at primary study level.			
	Nguyen	0.76	-0.30 to 1.81	95%	4	433	1	X		3
	Shepherd-Banigan	-0.32	-0.47 to -0.17	0%	6	707	4	1		1
Physical	Cramer	-0.65	-1.05 to -0.25	82%	9	718	Bias not reported at primary study level.			
	Nguyen	1.39	0.19 to 2.59	93%	3	333	X	X		3
Urogenital	Cramer	-0.53	-0.81 to -0.25	61%	7	661	Bias not reported at primary study level.			
Sexual	Nguyen	-0.36	-1.18 to 0.46	81%	2	150	X	X		2
**Mind-body therapies vs. no intervention**										
Total	Shorey	-1.51	-2.64 to -0.37	78%	2	92	X	1		1
**Resistance training vs. no intervention**										
Total	Shorey	-0.67	-1.51 to 0.18	NA	1	23	X	X		1
**Pelvic floor training vs. no intervention**										
Total	Nguyen	0.76	-0.40 to 1.92	78%	2	59	X	X		2
**Aerobic exercise vs. yoga**										
Total	Cramer	-0.21	-0.66 to 0.25	77%	4	386	Bias not reported at primary study level.			
Vasomotor	Cramer	-0.45	-0.87 to -0.04	72%	3	373	Bias not reported at primary study level.			
	Daley	-0.03	-0.45 to 0.38	61%	2	279	1	X		1
	Shepherd-Banigan (1 of 2 RCTs not combined within review)	-0.40	-0.78 to -0.02	NA	1	108	1	X		X
	Shepherd-Banigan (2 of 2 RCTs not combined within review)	-0.15	-0.41 to 0.11	NA	1	233	X	X		1
	Liu	0.15	-0.06 to 0.35	13%	3	432	1	1		1
Psychological	Cramer	-0.09	-0.47 to 0.28	76%	5	526	Bias not reported at primary study level.			
Physical	Cramer	-0.15	-0.55 to 0.26	79%	4	483	Bias not reported at primary study level.			
Urogenital	Cramer	-0.12	-0.53 to 0.3	74%	3	376	Bias not reported at primary study level.			
**Aerobic vs. resistance**										
Vasomotor	Sa	-2.60	-9.01 to 3.81	NA	1	36	1		X	X
Physical	Sa	-5.90	-16.17 to 4.37	NA	1	36	1		X	X
Psychological	Sa	-2.90	-8.24 to 2.44	NA	1	36	1		X	X
Sexual	Sa	0.40	-4.37 to 5.17	NA	1	36	1		X	X

## Comparison 1 – Exercise vs. no intervention

### Aerobic exercise vs. no intervention

#### Total menopause symptoms

Two recent, high-quality reviews [14, 46] assessed the effect of aerobic exercise on total menopause symptoms (seven RCTs, 542 participants). There is no primary study overlap between these high-quality reviews because of different inclusion criteria. One review [46] (two RCTs, 101 participants), showed a beneficial effect of aerobic exercise, the second review [14] (five RCTs, 441 participants) reported no effect of interventions.

#### Vasomotor symptoms

Four high-quality reviews reported vasomotor symptoms [6, 14, 46, 51] (21 RCTs, 2219 participants). Three reviews consistently reported no clear effect of aerobic exercise. Evidence from all three reviews [6, 14, 46] is impacted by imprecision, one review’s GRADE assessment was low certainty evidence [6]. One review separately analysed frequency and severity of vasomotor symptoms, finding no clear effect on frequency but a beneficial effect of aerobic exercise on severity [51].

#### Other symptoms

One high-quality review [14] found no clear effect for the four following symptom categories: psychological, physical, urogenital, sexual. For each outcome there was high heterogeneity, at least one study at high risk of bias, or both (Table 3).

### Exercise intensity: low

#### Psychological symptoms

Three reviews assessed low intensity exercise (including walking and yoga) [43, 44, 48] one was high-quality [48]. The high-quality review found no clear effect based on four RCTs (460 participants). The evidence was impacted by imprecision.

### Exercise intensity: moderate-high

#### Psychological symptoms

Three reviews assessed moderate to high intensity exercise for psychological symptoms [43, 44, 48] one was high-quality [48]. Moderate intensity exercise was consistently defined across all three reviews; aerobic and cardiovascular activity were considered moderately intense. The high-quality review found no clear effect based on seven RCTs (1250 participants).

### Exercise duration

Both ‘short to medium term exercise’ and ‘long term exercise’ versus no intervention were assessed by four reviews [43, 44, 46, 48] two were high quality [46, 48].

### Short to medium-term exercise (up to 6 months)

#### Total symptoms

One high-quality review [46] assessed between one and 24 weeks exercise and found a benefit based on five RCTs (282 participants).

#### Psychological symptoms

The second high-quality review [48] assessed up to 12 weeks of exercise for psychological symptoms and found a benefit based on seven RCTs (939 participants).

### Long-term exercise (over 6 months)

#### Psychological symptoms

One high-quality review [48] assessed long-term exercise for psychological symptoms (five RCTs, 1013 participants) and found no effect. The evidence is impacted by high risk of bias.

### Yoga versus no intervention

#### Total symptoms

One high-quality review [38] (eight RCTs, 671 participants) found a benefit on total menopause symptoms. This review included some studies at high risk of bias, but it was unclear how many such studies contributed to the effect.

#### Vasomotor symptoms

Three high-quality reviews [14, 38, 45] found differing results. One review [38] (eight RCTs, 548 participants), found a benefit of yoga. Two reviews [14, 45] (eleven RCTs and 383 participants) found no effect of interventions.

#### Psychological symptoms

Three high-quality reviews [14, 38, 45] included ten, four and six RCTs respectively when assessing the impact of yoga on psychological symptoms but the number of unique RCTs and participants is unclear. One review [45] showed a benefit of yoga; the other reviews found no clear effects [14, 38]. All reviews were impacted by high risk of bias in studies with substantial numbers of participants.

#### Physical symptoms

Two high-quality reviews [14, 38] (11 RCTs, unclear number of participants), found a benefit of yoga. Symptoms included were sleep, joint pain and fatigue. One review [38] included some high risk of bias studies but their relevance to this outcome is unclear.

#### Urogenital symptoms

One high-quality review [38] found a benefit of yoga from a meta-analysis of seven RCTs (661 participants). Review authors reported moderate heterogeneity (61%) and some high risk of bias.

#### Sexual symptoms

One high-quality review [14] found no effect of interventions from two RCTs (150 participants). This is strongly impacted by imprecision because of small numbers of participants.

### Mind-body therapy versus no intervention

#### Total symptoms

One high-quality review [46] found a positive effect favouring mind-body therapy based on two RCTs (92 participants). This review is impacted by high levels of imprecision because of the small number of participants.

### Resistance exercise versus no intervention

In both total and vasomotor symptoms evidence is very limited despite high-quality reviews.

#### Total symptoms

One high-quality review [46] included a single very small study (23 participants) which found no effect on total menopause symptoms. Very high levels of imprecision limit the certainty of this finding.

#### Vasomotor symptoms

One high-quality review [50] reported a benefit of resistance training on incidence of moderate or severe hot flash episodes. This is based on a single, very small RCT (58 participants) and the effect estimate reflects high levels of imprecision.

### Pelvic floor training versus no intervention

#### Total symptoms

One high-quality review [14] found no effect based on two RCTs (59 participants). Again, small numbers of participants mean evidence is very imprecise.

## Comparison 2: Different types of exercise compared with each other

### Aerobic exercise versus yoga

#### Total symptoms

One high-quality review [38] found no difference between aerobic exercise and yoga based on four RCTs (386 participants). While the review included some studies with high risk of bias it is unclear how many of these contributed to this evidence.

#### Vasomotor symptoms

Four high-quality reviews [6, 38, 45, 51] had mixed findings. One review [38] (three RCTs, 373 participants) found an effect in favour of yoga. This review included some studies at high risk of bias, but it was unclear how many contributed to the effect. Two reviews [6, 51] (two RCTs, 279 participants and three RCTs, 432 participants) each found no clear differences between aerobic exercise and yoga. A fourth review did not pool the two included RCTs (432 participants); one of these showed a benefit of yoga [45].

#### Other symptoms

One high-quality review [38] assessed aerobic exercise versus yoga on psychological (five RCTs, 526 participants), physical (four RCTs, 483 participants) and urogenital symptoms (three RCTs, 376 participants). There were no clear differences between the groups for any symptoms. This review included some high risk of bias studies, but it was unclear how many contributed to the effect estimates.

### Aerobic exercise versus resistance training

#### Total symptoms

One high-quality review [50] included one RCT (36 participants) comparing aerobic versus resistance training for 8 weeks. No differences were observed in total menopause quality-of-life domains. This result is subject to a high level of imprecision due to the small sample size.

#### Vasomotor symptoms

One high-quality [50] review reported an effect favouring resistance training over aerobic exercise on frequency of hot flash symptoms based on one RCT (36 participants). Due to the small sample size, this result is subject to a high level of imprecision.

## Discussion

This overview identified seventeen systematic reviews which included 80 relevant primary studies (8983 unique participants). Eight high-quality reviews evaluated various forms of physical activity. Fifteen reviews compared an activity to no intervention, eleven reviews researched aerobic exercise and eleven reviews researched yoga interventions, with limited evidence for other interventions. Nine reviews related to total symptom measures, vasomotor and psychological symptoms, with very limited evidence for physical, sexual, urogenital symptoms. Key limitations of the evidence from high-quality reviews were high risk of bias in primary studies, differences between review findings and heterogeneity between studies within reviews, and in some instances very high levels of imprecision due to few studies with very small numbers of participants. All of these reduce our confidence in the evidence.

Yoga was the intervention with most evidence (11 reviews) in terms of numbers of primary studies. There is evidence showing improvement of physical, urogenital, and total symptoms following yoga interventions two to three times weekly. Evidence for vasomotor and psychological symptoms was inconclusive. Session lengths and intervention durations varied, ranging from 20 to 120 mins and from three weeks to 24 months respectively. Findings for aerobic exercise were inconclusive although there were some examples of beneficial effects on total and vasomotor symptoms. Findings directly comparing yoga with aerobic exercise varied; it is unclear whether their effects differ. Evidence for resistance training was very limited. Based on the evidence synthesised in this overview, there is insufficient good quality evidence to recommend one form of exercise over any other. There is clear need for further high quality RCTs in this area to identify efficacious exercise interventions.

## Strengths and limitations

This overview systematically appraised the evidence for the effects of a wide range of physical activity on menopausal symptoms although interventions were predominantly structured forms of exercise rather than general activity. We used a comprehensive search and rigorous systematic review method; our synthesis focused on the highest-quality evidence. Identified reviews included primarily RCTs meaning most evidence originates from studies designed to answer questions of intervention efficacy. A risk with undertaking an overview is that included reviews may not be up to date with the most recent RCTs. An overview review also depends on existing systematic reviews including salient activities and outcomes. These limitations are to some degree mitigated by the relatively recent search dates and wide scope of included reviews, which should capture most interventions and outcomes. Limited impact was observed for our date and language restrictions: no reviews were excluded due to our post-2000 restriction; four reviews were excluded due to language limitations.

Overviews risk multiple counting of primary studies and participants due to overlap. Good reporting in included reviews allowed us to map overlap and determine the number of unique RCTs and participants contributing to each symptom assessment for most comparisons. This means we are confident we have not over-estimated the evidence base.

Overviews necessarily rely on the decisions and judgements of authors of included reviews which are sometimes poorly reported or unreliable. We have mitigated this by focusing on reviews assessed as high quality and by considering, wherever possible, the consistency and precision of their included evidence as well as risk of bias. We have noted where this was unclear. Activities were generally not well or systematically described (there was no use of TIDieR) [52] and many exercise interventions were simply classed as ‘aerobic’; further research should clearly delineate exercise categories to permit greater understanding of the impact of exercise type, intensity, and duration. A narrative review on walking as a potential benefit for menopause symptoms [53] previously highlighted this difficulty in navigating the variations in exercise duration and frequency as a challenge for researchers with respect to agreement on beneficial outcome.

Reporting of factors relating to equity was also limited, making the relevance of evidence to disadvantaged groups unclear. Current reviews categorise women on menopause status; broadening this to include ethnicity, income status, employment type and other PROGRESSPlus [32] factors will allow better understanding of the context in which an intervention is most effective.

Evaluation of HRT as a comparator was lacking; only seen in two reviews [6, 42]. Each review included a single RCT comparing this with exercise; findings were inconsistent with evidence strongly favouring HRT over physical activity for hot flashes [6] but a benefit of physical activity over HRT on menopause-specific quality of life was evidenced [42]. Reporting of complementary use of HRT was also limited. Since many women use exercise as an alternative to or complement of HRT, the comparison between and intersection of the two approaches could usefully be explored. HRT prescribing is unequal; in the UK women from lower socio-economic groups are around 30% less likely to be prescribed HRT than those from more affluent areas [54]. Communities with higher Black and minority ethnic populations are more likely to have lower socio-economic profiles, so multiple equity indicators are likely involved in prescribing [55, 56]. Strengthening evidence for all menopause management options available to women may support equity and accessibility of useful treatments.

## Further research

Reviews frequently reported there was no evidence of a difference between exercise groups and controls, or between different types of exercise, but it should be noted that most included RCTs were underpowered. Thus findings of no difference between different types of exercise do not indicate that either exercise would have no effect relative to no intervention; but an assessment of appropriate transitivity would need to be undertaken in order proceed with a network meta-analysis of all available trials to fully utilise the data for this. Direct comparisons indicate that both yoga and aerobic exercise may sometimes be effective relative to no intervention. In many cases included trials were too small and too few to identify effects; optimal information sizes were not calculated but would likely not be met. It is therefore important that reviews, and our synthesis, are not interpreted as evidence of no effect; but rather as evidence that more adequately powered studies are needed.

We have identified several research gaps via this overview. More research on resistance training and high intensity aerobic exercise is needed, as is research on the impact of exercise on physical, sexual, and urogenital symptoms. Beyond this, a comprehensive review and network meta-analysis would enable full use of all the available RCT evidence to identify where new trials are most urgently needed. Any future RCTs should be adequately powered, rigorously designed, and clearly reported. This includes clear description of interventions using TIDieR domains [52] and clear reporting of PROGRESSPlus participant characteristics [32].

## Conclusion

There is some evidence that both yoga, and to a lesser extent, aerobic exercise may be beneficial for some menopause symptoms, but there is insufficient evidence to recommend a particular form of exercise for specific symptom management. Exercise benefits for general health are well documented; both aerobic and resistance training are generally recommended [9]. These recommendations, together with the personal preferences of women, are currently likely to be most important in guiding exercise choice and clinical prescription.

### Electronic supplementary material

Below is the link to the electronic supplementary material.

Supplementary Material 1

Supplementary Material 2

## Data Availability

This overview includes only previously published data. Further information on aspects of the overview process not included in the supplementary information (I.e., lists of excluded studies) are available on request from the authors; please email Annemarie Money annemarie.money@manchester.ac.uk.
