# Supplementary material for: The impact of physical activity and exercise interventions on symptoms for women experiencing menopause: overview of reviews
Source: BMC Womens Health. 2024 Jul 13;24:399. doi: 10.1186/s12905-024-03243-4 (PMC11245773; doi:10.1186/s12905-024-03243-4)
Supplement: Supplementary file 2 — Supplementary Material 2 [file 12905_2024_3243_MOESM2_ESM.docx]

**Supplementary Table 1 Summary of Findings (moderate, low and critically low-quality reviews)**

| **Outcome** | **Review** | **Effect estimate/narrative summary** | **RCTs** | **Participants** | **Risk of bias** | | |
| --- | --- | --- | --- | --- | --- | --- | --- |
|  |  |  |  |  | **High** | **Low** | **Unclear** |
| **Aerobic exercise vs no intervention** | | | | | | | |
| Total | Kalra | 2 RCTs found an improvement in overall menopause symptoms. 2 RCTs showed improved QoL. | 4 | 378 | 4 | X | X |
|  | Carcelén-Fraile | 2 RCTs found an improvement in overall menopause symptoms based on MENQOL and KMI total score. | 2 | 512 | 2 | X | X |
| Vasomotor | Carcelén-Fraile | 1 RCT found an effect and 1 pre-post study found no effect on the MENQOL vasomotor domain. | 2 | 374 | 1 | The pre-post study was not assessed. | |
|  | Woods | 1 RCT found a decrease in vasomotor symptoms. 5 RCTs found no significant effect on vasomotor symptoms following an exercise intervention. 1 RCT found an increased risk of moderate-severe hot flashes. | 7 | 1046 | Risk of bias or methodological quality not assessed. | | |
| Psychological | Kalra | 6 RCTs reported an improvement in psychological health. | 6 | 562 | 6 | X | X |
|  | Woods | 2 reported improvements in anxiety and mental health score. 1 showed reduced risk of memory problems but no significant change in depressive symptoms. 1 improvement in mental health. 2 studies reported no significant effect, 1 study showed no improvement in general mood compared to hormone therapy. | 6 | 882 | Risk of bias or methodological quality not assessed. | | |
| Physical | Carcelén-Fraile | 3 RCTs found an improvement in physical symptoms. 1 RCT specifically mentions an improvement in fatigue, joint pain and headaches. | 3 | 934 | 3 | X | X |
|  | Woods | 1 study reported improved body pain. 1 RCT reported no significant difference of somatic severity. 1 reported no significant change to physical impact scores. 2 studies reported improved sleep quality, however a third reported not significant. | 5 | 678 | Risk of bias or methodological quality not assessed. | | |
| Sexual | Carcelén-Fraile | 1 pre-post study reported no significant change in sexual function.  2 RCTs did show improvements and 1 RCT did not show improvement in sexual symptoms. | 4 | 563 | 2 | Two studies were not assessed. | |
| **Low intensity exercise vs no intervention** | | | | | | | |
| Psychological | Perez-Lopez | SMD -0.41 (95% CI -0.63 to -0.19) | 7 | 1241 | 7 | X | X |
|  | Nigdelis | SMD -0.54 (95% CI -0.71 to -0.35) | 1 | 535 | 1 | X | X |
| **Moderate intensity exercise vs no intervention** | | | | | | | |
| Psychological | Perez-Lopez | SMD -0.31 (95% CI -0.56 to -0.05) | 4 | 743 | 4 | X | X |
|  | Nigdelis | SMD -0.08 (95% CI -0.33 to 0.17) | 4 | 1011 | 4 | X | X |
| **Short to medium term exercise vs no intervention** | | | | | | | |
| Psychological | Perez-Lopez | SMD -0.44 (95% CI -0.69 to -0.18) | 6 | 809 | 6 | X | X |
|  | Nigdelis | SMD -0.17 (95% CI -0.59 to 0.25) | 3 | 471 | 3 | X | X |
| **Long term exercise vs no intervention** | | | | | | | |
| Psychological | Perez-Lopez | SMD -0.29 (95% CI -0.49 to -0.09) | 5 | 1175 | 5 | X | X |
|  | Nigdelis | SMD -0.02 (95% CI -0.42 to 0.38) | 3 | 491 | 3 | X | X |
| **Yoga vs no intervention** | | | | | | | |
| Total | Innes | 2 non-randomised control trials improved total symptoms between 35.7% and 58.2% post-intervention. | 2 | 26 | Risk of bias or methodological quality not assessed. | | |
|  | Lee | SMD 0.07 (95% CI -0.25 to 0.39) | 2 | 157 | Risk of bias or methodological quality not assessed. | | |
|  | Carcelén-Fraile | 1 RCT showed an improvement in total symptoms. | 1 | 355 | 1 | X | X |
| Vasomotor | Innes | All studies saw some improvement in vasomotor symptoms however these ranged widely from 5.8% to 71.1%. | 6 | 221 | Risk of bias or methodological quality not assessed. | | |
|  | Carcelén-Fraile | 1 RCT showed an improvement in MENQOL vasomotor score and daily hot-flash interferences. | 1 | 355 | 1 | X | X |
| Psychological | Innes | 3 studies found an improvement in psychological and psychosocial symptoms. | 3 | 192 | Risk of bias or methodological quality not assessed. | | |
| Physical | Innes | 3 studies found an improvement in somatic and physical symptoms. | 3 | 192 | Risk of bias or methodological quality not assessed. | | |
| Sexual | Carcelén-Fraile | 1 RCT showed improvements in MENQOL sexual domain. | 1 | 355 | 1 | X | X |
| **Mind-body therapies vs no intervention** | | | | | | | |
| Vasomotor | Innes | 2 studies found Tai Chi and active mindfulness classes improved vasomotor symptoms and hot flash frequency. | 2 | 76 | Risk of bias or methodological quality not assessed. | | |
|  | Carcelén-Fraile | 1 RCT had improvements in MENQOL vasomotor domain (p=0.040) following a Rusie Dutton intervention. | 1 | 54 | 1 | X | X |
| Psychological | Innes | 1 non-randomised control trial found a 32.5% improvement in psychosocial symptoms following active mindfulness classes. | 1 | 18 | Risk of bias or methodological quality not assessed. | | |
|  | Kalra | 1 RCT reported improvements in psychological health following a Pilates intervention. | 1 | 110 | 1 | X | X |
|  | Carcelén-Fraile | 1 RCT had improvements in MENQOL psychological domain (p=0.000) following a Rusie Dutton intervention. | 1 | 54 | 1 | X | X |
| Physical | Innes | 1 RCT found a 17.8% improvement in physical symptoms. | 1 | 18 | Risk of bias or methodological quality not assessed. | | |
|  | Carcelén-Fraile | 1 RCT had improvements in MENQOL physical domain (p=0.000) following a Rusie Dutton intervention. | 1 | 54 | 1 | X | X |
| Sexual | Carcelén-Fraile | 1 RCT had improvements in MENQOL psychological domain (p=0.003) following a Rusie Dutton intervention. | 1 | 54 | 1 | X | X |
| **Resistance training vs no intervention** | | | | | | | |
| Total | Kalra | 1 RCT found an improvement in QoL. | 1 | 39 | 1 | X | X |
|  | Capel-Alcaraz | 2 RCTs measured QoL but results were not reported within the review. | 2 | 274 | 1 | 1 | X |
| Vasomotor | Capel-Alcaraz | 1 RCT found significant improvements in hot flashes following exercise with resistance bands. | 1 | 20 | X | 1 | X |
| Physical | Capel-Alcaraz | 1 RCT found a significant improvement in hip pain compared to control group following strength training at 12 and 52 weeks. | 1 | 94 | 1 | X | X |
| **Pelvic floor training vs no intervention** | | | | | | | |
| Sexual | Carcelén-Fraile | 2 RCTs found an effect on sexual symptoms | 3 | 422 | 3 | X | X |
| **Aerobic exercise vs yoga** | | | | | | | |
| Total | Innes | 1 RCT reported no significant change in total menopause symptoms | 1 | 164 | Risk of bias or methodological quality not assessed. | | |
| Vasomotor | Innes | 2 RCTs found an improvement in vasomotor symptoms. | 2 | 284 | Risk of bias or methodological quality not assessed. | | |
|  | Lee | SMD 0.34 (95% CI -0.27 to 0.96) | 2 | 232 | Risk of bias or methodological quality not assessed. | | |
| Psychological | Innes | 2 RCTs found an improvement in psychosocial symptoms ranging from 12.5% to 40.9% improvement. | 2 | 284 | Risk of bias or methodological quality not assessed. | | |
|  | Lee | SMD 0.72 (95% CI -1.67 to 3.11) | 2 | 232 | Risk of bias or methodological quality not assessed. | | |
| Physical | Innes | 2 RCTs found an improvement in sleep disturbances. | 2 | 284 | Risk of bias or methodological quality not assessed. | | |
|  | Lee | SMD -0.20 (95% CI -1.22 to 0.82) | 2 | 232 | Risk of bias or methodological quality not assessed. | | |

**Supplementary Table 2. Details of AMSTAR questions and ratings for each included review**

| AUTHOR | Q1 | Q2 | Q3 | Q4 | Q5 | Q6 | Q7 | Q8 | Q9A | Q9B | Q10 | Q11 | Q12 | Q13 | Q14 | Q15 | Q16 | OVERALL RATING |
| --- | --- | --- | --- | --- | --- | --- | --- | --- | --- | --- | --- | --- | --- | --- | --- | --- | --- | --- |
| CAPEL-ALCARAZ | Y | Y | N | PY | Y | Y | PY | Y | Y | NA | Y | NA | NA | N | Y | NA | Y | Low |
| CARCELÉN FRAILE | Y | PY | N | PY | Y | Y | PY | PY | Y | NA | N | NA | NA | N | N | NA | Y | Low |
| CRAMER | Y | PY | Y | PY | Y | Y | PY | Y | Y | NA | N | Y | Y | Y | Y | Y | Y | High |
| DALEY | Y | Y | Y | Y | Y | Y | Y | Y | Y | NA | N | Y | Y | Y | Y | Y | Y | High |
| INNES | Y | Y | Y | PY | Y | Y | PY | Y | Y | PY | N | NA | NA | Y | Y | NA | Y | Moderate |
| KALRA | Y | PY | N | PY | N | Y | N | PY | Y | NA | N | NA | NA | N | N | NA | Y | Critically low |
| LEE | N | PY | Y | PY | Y | Y | PY | Y | PY | NA | N | Y | Y | Y | Y | N | N | Low |
| LIU | Y | Y | Y | PY | Y | Y | PY | Y | Y | NA | N | Y | Y | Y | Y | Y | Y | High |
| MARTINEZ DOMINGUEZ | Y | PY | Y | PY | Y | Y | PY | Y | Y | NA | N | Y | Y | Y | Y | Y | Y | High |
| NEDROW | Y | PY | N | PY | Y | Y | PY | Y | PY | NA | N | NA | NA | Y | Y | NA | Y | Moderate |
| NGUYEN | Y | PY | Y | Y | Y | Y | PY | Y | Y | NA | N | Y | Y | Y | Y | Y | Y | High |
| NIGDELIS | Y | PY | N | Y | Y | Y | PY | Y | Y | NA | N | Y | N | N | Y | N | Y | Critically low |
| PEREZ LOPEZ | Y | PY | N | PY | Y | Y | PY | Y | Y | NA | N | Y | Y | N | Y | Y | Y | Low |
| SA | Y | Y | N | Y | Y | Y | Y | Y | Y | NA | Y | Y | Y | Y | Y | Y | Y | High |
| SHEPHERD BANIGAN | Y | Y | Y | PY | Y | Y | PY | Y | Y | NA | N | Y | Y | Y | Y | Y | Y | High |
| SHOREY | Y | PY | Y | Y | Y | Y | PY | PY | Y | NA | N | Y | Y | Y | Y | Y | Y | High |
| WOODS | Y | PY | Y | PY | Y | Y | N | Y | N | NA | N | NA | NA | N | N | NA | Y | Critically low |

Q1 - Did the research questions and inclusion criteria for the review include the components of PICO? Q2 - Did the report of the review contain an explicit statement that the review methods were established prior to the conduct of the review and did the report justify any significant deviations from the protocol? Q3 - Did the review authors explain their selection of the study designs for inclusion in the review? Q4 - Did the review authors use a comprehensive literature search strategy? Q5 - Did the review authors perform study selection in duplicate? Q6 - Did the review authors perform data extraction in duplicate? Q7 - Did the review authors provide a list of excluded studies and justify the exclusions? Q8 - Did the review authors describe the included studies in adequate detail? Q9 - Did the review authors use a satisfactory technique for assessing the risk of bias (RoB) in individual studies that were included in the review? Q10 - Did the review authors report on the sources of funding for the studies included in the review? Q11 - If meta-analysis was performed did the review authors use appropriate methods for statistical combination of results? Q12 - If meta-analysis was performed, did the review authors assess the potential impact of RoB in individual studies on the results of the meta-analysis or other evidence synthesis? Q13 - Did the review authors account for RoB in individual studies when interpreting/ discussing the results of the review? Q14 - Did the review authors provide a satisfactory explanation for, and discussion of, any heterogeneity observed in the results of the review? Q15 - If they performed quantitative synthesis did the review authors carry out an adequate investigation of publication bias (small study bias) and discuss its likely impact on the results of the review? Q16 - Did the review authors report any potential sources of conflict of interest, including any funding they received for conducting the review? Y – Yes PY – Partial Yes N – No

**Supplementary Table 3 example search Medline**

| #1 | 'menopause and climacterium'/exp OR 'menopause':ti,ab OR 'menopausal':ti,ab OR 'peri-menopause':ti,ab OR 'peri-menopausal':ti,ab OR 'perimenopause':ti,ab OR 'perimenopausal':ti,ab OR postmenopause:ti,ab OR postmenopausal:ti,ab OR post-menopause:ti,ab OR post-menopausal:ti,ab OR climacteric:ti,ab OR 'hot flash':ti,ab OR 'hot flashes':ti,ab OR 'hot flush':ti,ab OR 'hot flushes':ti,ab OR 'night sweat':ti,ab OR 'night sweats':ti,ab OR 'vasomotor symptoms':ti,ab |
| --- | --- |
| #2 | ‘systematic review’:ti,ab |
| #3 | 1 AND 2 |
| #4 | Publication Date from 2000/01/01 to 2023/06/01 |
| #5 | 3 AND 4 |
| #6 | 'exercise'/exp OR 'sport'/exp OR 'Resistance Training':ti,ab OR 'Exercise':ti,ab OR 'Exercises':ti,ab OR ‘exercise movement techniques’:ti,ab OR ‘sport’:ti,ab OR 'physical activity':ti,ab OR ‘physical fitness’:ti,ab OR 'aerobic activity':ti,ab |
| #7 | 5 AND 6 |
